# Factors Affecting Adherence to Antiretroviral Treatment in Harari National Regional State, Eastern Ethiopia

**DOI:** 10.1155/2013/960954

**Published:** 2013-08-29

**Authors:** Habtamu Mitiku, Tekabe Abdosh, Zelalem Teklemariam

**Affiliations:** ^1^Department of Medical Laboratory Sciences, College of Health and Medical Sciences, Haramaya University, P.O. Box 235, Harar, Ethiopia; ^2^College of Health and Medical Sciences, Haramaya University, School of Medicine, P.O. Box 235, Harar, Ethiopia

## Abstract

*Background*. The efficacy of antiretroviral treatment (ART) depends on strict adherence to the regimen, but many factors have been identified for nonadherence. *Method*. To identify the factors for non-adherence to ART, a cross-sectional study was conducted on people living with human immunodeficiency virus (HIV) and attending the ART service at Hiwot Fana and Jugal hospitals; it was done from October to December, 2010. Adherence was defined as taking 95% of the prescribed doses in the week before the survey. Data were collected using a standard interview questionnaire and were analyzed using SPSS Version 16. *Result*. Among the 239 study participants, the magnitude of adherence to ART in the week before interview was 87%. The main reasons for nonadherence were forgetting (47.2%), traveling (18.9%), and being busy doing other things (15.1%). There was not any independent predicator identified for adherence to ART. *Conclusion*. Compared to other similar studies in Ethiopia, in this study a high adherence rate was found. Forgetfulness was the most common reason for the nonadherence. Therefore, the ART counseling needs to give emphasis to using memory aids. In addition, a further study on adherence rate and its determinants with multiple adherence measurements is recommended.

## 1. Background

HIV/AIDS is the greatest health problem in the world. At the end of 2009, for example, an estimated 33.3 million people were living with HIV, 2.6 million people were newly infected, and 1.8 million lost their lives due to AIDS. Sub-Saharan Africa carries 68% of the global total HIV burden (22.5 million people), which is an inordinate share [1].

ART increases the length, quality of life, and productivity of the people living with HIV (PLWH) by improving survival and decreasing the incidence of opportunistic infections through reduction of the viral load and increase of the level of CD_4_ cells [2]. Cognizing this, the government of Ethiopia has done a lot to introduce the treatment [3]. It was first offered in July 2003 through 12 government hospitals on a copayment basis. In early 2005, 211.000 men, women, and children needed ART but only 16,400 received it. In January 2005, free ART through the Global Fund, World Bank, and US President's Emergency Plan for AIDS Relief (PEPFAR) became available in 22 hospitals [4]. There were 497 ART centers serving 167.271 HIV/AIDS patients in October 2009 [5].

The effectiveness of ART relies on a strict adherence to it. In other words, loose obedience, or non obedience to ART can result in inadequate viral suppression, immunologic failure, rapid disease progression, and the development of drug resistance [6, 7]. To avoid the emergence of the resistant strains of the virus, World Health Organization (WHO) recommends at least 95% of adherence to ART [8]. Based on these facts, the importance of adhering to ART has been widely publicized and accepted as a critical element in the success of ART.

However, many of the reported adherence rates are below the recommendation. Among the patients in Soweto, South Africa, the magnitude of adherence was 88% [9]; in a community setting in Atlanta [10], twenty percent of the participants missed at least one dose of ART. In Ethiopia, an adherence rate of 83% was reported in two hospitals of Oromiya Regional State [11], 88.3% in Yirgalem Hospital [12], and 81.2% in three hospitals in Addis Ababa [13].

The commonly identified factors for nonadherence are forgetfulness, poor understanding of the relationship between nonadherence and disease progression, side effects of drugs, alcohol and drug abuse, poor social support, poor health provider-patient relationships, being away from home, fear of disclosure, educational level, and others [12–15]. We, therefore, aimed at determining the level of adherence and identifying the factors that are related to nonadherence to ART among the patients attending at ART units in Harari National Regional State, eastern Ethiopia.

## 2. Methods

### 2.1. Study Area, Design, and Period

The study was done in Harari National Regional State. It is found in eastern Ethiopia, and it is 525 km from Addis Ababa. In the region, ART was started in 2005, and there were 1876 people on ART in 2009 (Harari National Regional State Health Report). A cross-sectional study was conducted in the ART units of Hiwot Fana Hospital and Jugal Hospital from October to December, 2010. The PLWH who were attending at the ART unit of either hospital, who were more than 18 years old, who had taken antiretroviral medication at least for one month, and who gave their signed consent to participate in the study, were included.

### 2.2. Sample Size and Sampling Techniques

In this study the sample size was calculated by using the 0.83 proportion of adherence in the two hospitals of Oromiya Regional Sate [11], 5% margin of error, and 95% confidence interval. Then, 10% nonresponse rate was added. The total sample size was 239. This was allotted to the hospitals proportionally to their monthly average client flow. Finally, by using a systematic sampling method, we selected 160 and 79 ART unit clients from Hiwot Fana Hospital and Jugal Hospital, respectively.

### 2.3. Data Collection

The data were collected using a structured questionnaire. The questionnaire contained different variables such as sociodemographic characteristics, social support, information related to personal life, alcohol/substance use, treatment side effects, patient self-report for treatment adherence, patient reported reasons for nonadherence, and others. The participants' CD_4_ T-cell count before starting and the most recent CD_4_ count after starting ARV drugs were retrieved from their ART follow-up records in the hospitals. Nurses who were trained on ART but not currently working in the ART units collected the data.

### 2.4. Data Analysis

The data were analyzed using SPSS Version 16 software. Adherence to ART in the previous seven days of the interview was measured by the self-report about taking pills. The self-reported dose adherence was defined as a respondent's self-report whether any antiretroviral medication had been skipped in the previous seven days. A respondent was said to be adherent if he/she took ≥95% of the prescribed doses in the previous week. The comparison of those who were adherent (≥95% of their doses of ART medication taken) and nonadherent (≤95% of their doses of ART medication taken) was carried out with various variables. The predicators of adherence were assessed using bivariate logistic regression model. Chi-square test was used to assess the difference between CD_4_ count before starting and the most recent CD_4_ count after starting ARV drugs. 95% confidence interval with *P* value ≤ 0.05 was considered as statistically significant. Since no predicator variables were found statistically significant with outcome variable using bivariate logistic regression model, multivariate logistic regression model was not employed to identify the independent predictor of adherence (outcome variable).

### 2.5. Ethical Considerations

Ethical clearance was obtained from Haramaya University Colleges of Health and Medical Sciences Institutional Research and Ethical Review Committee. All the participants were given an explanation about the objective of the study and their right to participate or not to participate in the study. Those who gave their signed written consent participated. ART follow-up records were retrieved by the nurses working in the ART units, and personal information of the participants was kept confidential.

## 3. Results

### 3.1. Sociodemographic Information

Of the 239 study participants, 73.6% were female, 72.4% were between 20 and 39 years of age, 42.7% were married, and 87.4% were literate (Table 1). Their average age was 35 ± 8.69 years with a range of 20 to 69.

### 3.2. Factors Related to Personal Life and Social Support

About 92.1% and 7.9% of the participants were ≤5 years and >5 years on ART, respectively, with a mean duration of 3 years (range of 1 month to 7 years). Almost all of the respondents (98.3%) took ≤5 pills per day, with a mean of 2.8 pills. About 17.2% of the participants used active substances (Table 2).

Many of the participants (70.7%) lived with other people (spouse, children, parents, siblings, other relatives, and friends). About 47.3% of them had financial, psychological, and physical support from their families. Only 14.2% had financial, psychological, physical, and other support from outside their families (friends, nongovernmental organizations, community-based organizations, religious based organizations, governmental facilities, and others). Many participants had disclosed their HIV status to their family or others (78.7%). They had known their HIV status for a mean of 5 years, with a range of 3 months to 17 years. Of the 73.2% of the participants who had children, 13.4%, 83.3%, and 3.3% of their children were seropositive, seronegative, and not aware of their serostatus, respectively (Table 2).

### 3.3. Adherence Rate of ART

Two hundred eight (87%) of the participants had taken >95% of their prescribed ARV drugs for the past 7 days. Almost similar adherence rate was observed among males (85.7%) and females (87.5%). Higher adherence rate was observed in the age group of 20–30 years (92.8%). However, adherence rate was not statistically associated with sex (COR: 1.17; 95 CI: 0.51, 2.69) and age group (COR: 1.46; 95 CI: 0.98, 2.17) (Table 1). 

Higher adherence rate (90.2%) was observed among singles compared to married study participants (84.3%). The respondents who had high school and above education were more adhered (90.7%) than those who were illiterate (80%). However, there was no statistically significant association between the adherence rate and the marital status and the educational level of the study participants. Higher adherence rate, 88.1% and 97.6%, was observed among employed and study participant's earning ≥500 birr per month, respectively. But no significant statistical association was found between the adherence rate, the occupational status (COR: 1.731; 95 CI: 0.69, 4.37), and the economic status (COR: 5.49; 95 CI: 0.69, 43) of the study participants (Table 1).

Adherence rate of 86.7% was reported among patients who had support from their family while 88.2% adherence rate reported among patients who had support from friends, governmental facilities, nongovernmental organizations, community-based organizations, and religious organizations. Adherence rate of 87.2% and 86.3% was reported among patients who disclosed their serostatus for families, friends, and others and who did not disclose their serostatus, respectively. About 92.7% and 85.9% adherence rates were reported by patients who were using active substances and who were not using active substances, respectively. Social support, disclosure of serostatus, and substance abuse did not have significant influence on the adherence rate of the participants (Table 2).

Adherence rate of 87.3% and 84.2% was reported by the respondents who took the treatment for ≤5 and >5 years, respectively. About 86.5% and 100% adherence rates were reported by the study participants who took ≤5 and >5 pills per day, respectively. Neither the number of years on treatment nor the pill uptake per day had a significant influence on adherence rate of the participants (Table 2).

The CD_4_ counts of, before starting ART, 224 (93.3%) participants were found, and 164 (73.2%) of them had CD_4_ count <200 cells/mm^3^, with a mean of 155.6 cells/mm^3^. The most recent CD_4_ counts of 207 (86.6%) participants were surveyed, and only 17 (8.2%) of them had CD_4_ count <200 cells/mm^3^, with a mean of 443.3 cells/mm^3^. A significant increase in CD_4_ cell count was observed after the initiation of the treatment (*P* < 0.01) (Table 3).

### 3.4. Reasons for Nonadherence

**Figure 1:**
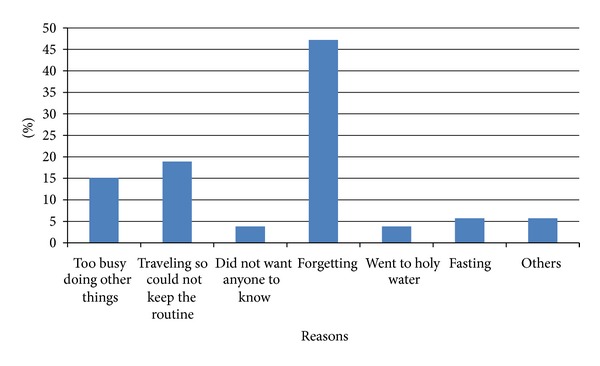
Reasons for missing ART medications by study participant in Hiwot Fana and Jugal hospital, 2010.

Few participants (13%) did not adhere to ART. The average number of doses missed in the past 7 days was 1.3. Some missed the treatment due to forgetting (47.2%), some due to travelling (18.9%), and few due to being busy (15.1%) (Figure 1).

### 3.5. Side Effects of ART Reported by the Respondents

**Figure 2:**
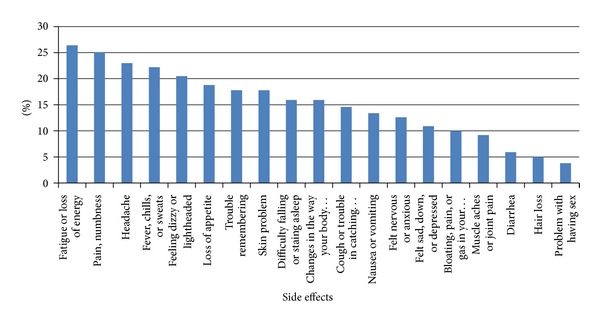
Side effects of ART reported by study participants in Hiwot Fana and Jugal Hospitals, 2010.

Many of the participants (73.3%) reported one or more side effects of ARV drugs. The most common side effects reported were fatigue or loss of energy (26.4%) and numbness of tingling in hands and feet (25.1%) (Figure 2). All most equal adherence rate, 73% and 74.2%, was reported by the study participants who reported one or more treatment side effects and who did not, respectively. There was no significant association between the adherence rate and the presence or absence of treatment side effects (COR: 0.94; 95 CI: 0.39, 2.23).

## 4. Discussion

The adherence rate in the study area was 87%. This was comparable with the one found in Yirgalem Hospital (88.3%) [12] and Soweto, South Africa, (88%) [9]. But it was higher than the rate reported from Oromiya Regional State (83%) [11], Addis Ababa (81.2%) [13], and other developing countries (ranges from 40% to 70%) [14, 16]. The difference might be due to activists which have been undertaken to improve the ART service by governmental organizations and NGOs working in the area. 

Tablet burden can often affect any medication adherence. A previous study explained that the fundamental challenge to ART is the complexity regimen, which may include >20 pills per day [14]. Due to the advancement in ART drugs, the daily dose is being reduced [17]. The participants in this study reported that they took a maximum of 7 pills per day; this may have contributed to the high rate of their adherence [11, 13].

 Age, sex, and marital status did not significantly affect the adherence rate of the participants. This is also reported by other studies elsewhere [16, 18, 19]. The income of the participants did not significantly affect their adherence. This may be due to the fact that ARV treatment has been given free of charge in Ethiopia since 2005 through the Global Fund, World Bank, and US President's Emergency Plan for AIDS Relief (PEPFAR) [4]. Low economic status was not a predictor of adherence for patients with fully subsidized therapy [12, 17, 20]. A study conducted on Myanmar migrants living in Thailand also showed that free-of-charge treatment strongly improves a long-term regular ART adherence [21].

Like the report by Cauldbeck et al. and Amberbir et al. [17, 20], in this study, although the adherence rate of the respondents increased with an increase in their educational level, there was no significant association between the adherence rate and the educational level. Better educated generally have access to information and are more likely to make better informed decisions [22]. Verbal instructions to patients who are illiterate seem equally as effective as written instructions which are given to all patients [17]. 

Studies showed that support from family and others supporting PLWH were predictors of ART adherence [14, 19, 20]. In this study, 47.3% of the respondents had family support while only 14.2% had support from friends, community, government, NGOs, and other groups supporting PLWH. And those study participants who had family support less adhered to the treatment than those who had no family support. Even though patients have family support they may miss the dose in working places and other places outside home. This implicates that family support alone is not a factor to increase the uptake of ARV; support from friends, community, government, NGOs, and other groups supporting PLWH also needs to be emphasized.

The respondents' adherence rate was inversely proportional to the length of time they had been on ART. That is, the longer they were on ART, the lesser they adhered. A similar trend was found from studies done in Africa, in the early therapy, before they develop the long term adverse effects of the therapy, HIV positives adhere more because they experience a dramatic increase in their health status [23].

 The study participants who disclosed their HIV serostatus adhered to the treatment (87.2%) more than those who did not disclose (66.3%). The latter were likely to have a frequent treatment interruption due to the fact that tablets must be hidden and therefore not be taken in the presence of others [23]. So encouraging voluntary HIV status disclosure in a community with access to ART may help to decrease stigma and improve adherence.

Those that experience side effects from their medications are known to be risk patients for nonadherence to ART [14]. But this was not found to have a significant influence on adherence in this study. This may be due to the mildness of the side effects reported. Those experiencing milder side effects such as skin rash or skin discolorations, fatigue, headache, and fever were more adherent to ART than those experiencing more severe side effects such as metabolic effects (CNS toxicity, sever hepatic necrosis, and renal toxicity) [17].

Forgetfulness, traveling, and being too busy were the most common reasons for poor adherence to medications in this study. Similar reasons were reported in studies conducted in Adama and Jimma hospitals [11] and Yirgalem Hospital [12], Ethiopia, and in Kenya [18].

There is no gold standard for measuring adherence, and our measurement of adherence is only based on patients' report on missed doses; this may be subjected to social desirability and recall bias, and literature showed that patients tend to overestimate adherence [24]. So the small overall number of nonadherent patients could have interfered with a significance of the statistical difference found.

## 5. Conclusion

In general, in this study relatively a higher adherence rate was reported compared to other studies in Ethiopia. Forgetfulness was the most common reasons for poor adherence to the medication. 

A study conducted in southwest Ethiopia showed that patients who use memory aids were three times more likely to be adherent than those who did not [20]. Therefore, adherence counseling and health information dissemination need to include strategies to minimize forgetfulness using memory aids such as pill boxes, written schedule, and watch bell. In addition, a further study on adherence rate and its determinants with multiple adherence measurements to resolve the barriers to adherence is also recommended.

## Figures and Tables

**Table 1:** Adherence rate by sociodemographic characteristics of the study participants at Hiwot Fana and Jugal hospitals, 2010.

Sociodemographic variable	Adherence status		Total	COR (95 CI)
	Adhered number (%)	Not adhered number (%)		
Sex				
Male	54 (85.7)	9 (14.3)	63 (26.4)	1.00
Female	154 (87.5)	22 (12.5)	176 (73.6)	1.17 (0.51, 2.69)
Age				
20–30	77 (92.8)	6 (7.2)	83 (34.7)	1.00
31–39	77 (85.6)	13 (14.4)	90 (37.7)	0.36 (0.08, 1.63)
40–49	40 (81.6)	9 (18.4)	49 (20.7)	0.79 (0.20, 3.13)
≥50	14 (82.4)	3 (17.6)	17 (7)	1.05 (0.25, 4.44)
Marital status				
Married	86 (84.3)	16 (15.7)	102 (42.7)	1.00
Single	37 (90.2)	4 (9.8)	41 (17.2)	1.49 (0.51–4.35)
Divorced	45 (88.2)	6 (11.8)	51 (21.3)	0.87 (0.22, 3.47)
Widowed	40 (88.9)	5 (11.1)	45 (18.8)	1.07 (0.30, 3.76)
Educational level				
High school and above	78 (90.7)	8 (9.3)	86 (36)	1.00
Regular school 1–8	99 (86.1)	16 (13.9)	115 (48.1)	1.58 (0.64, 3.87)
Able to read and write	7 (87.5)	1 (12.5)	8 (3.3)	1.40 (0.15, 12.8)
Unable to read and write	24 (80)	6 (20)	30 (12.6)	2.44 (0.77, 7.72)
Occupation				
Employed	178 (88.1)	24 (11.9)	202 (84.5)	1.00
Unemployed	30 (81.1)	7 (18.9)	37 (15.5)	1.73 (0.69, 4.37)
Income				
00	40 (97.6)	1 (2.4)	41 (17.2)	1.00
≤500	102 (87.9)	14 (12.1)	116 (48.5)	5.49 (0.69, 43.1)
Do not know*	66 (80.5)	16 (19.5)	82 (34.3)	

*66 participants do not know their average monthly income and are not included in income analysis.

COR: crude odds ratio.

CI: confidence interval.

**Table 2:** Adherence rate by social support, HIV status disclosure, and substance abuse. Duration of ART treatment and pill burden of the study participants at Hiwot Fana and Jugal hospitals, 2010.

Variable	Adherence status		Total number (%)	COR (95 CI)
	Adhered number (%)	Not adhered number (%)		
Substance abuse				
No	170 (85.9)	28 (14.1)	198 (82.8)	1.00
Yes	38 (92.7)	3 (7.3)	41 (17.2)	0.48 (0.14, 1.66)
Duration				
≤5 year	192 (87.3)	28 (12.7)	220 (92.1)	1.00
>5 years	16 (84.2)	3 (15.8)	19 (7.9)	0.78 (0.21, 2.84)
Support from family				
Yes	98 (86.7)	15 (13.3)	113 (47.3)	1.00
No	110 (87.3)	16 (12.7)	126 (52.7)	1.05 (0.49, 2.23)
Support from others				
Yes	30 (88.2)	4 (11.8)	34 (14.2)	1.00
No	178 (86.8)	27 (13.2)	205 (85.8)	0.88 (0.29, 2.69)
Disclosure of serostatus				
Yes	164 (87.2)	24 (12.8)	188 (78.7)	1.00
No	44 (86.3)	7 (13.7)	51 (21.3)	0.92 (0.37, 2.28)

COR: crude odds ratio.

CI: confidence interval.

**Table 3:** CD_4_ cell count before and after initiation of ART treatment among the study participants at Hiwot Fan and Jugal hospitals, 2010.

CD_4_ count	Before treatment No. (%)	After treatment No. (%)	*P* value
<200 cell/mm^3^	164 (73.2)	17 (8.2)	<0.01
200–499 cells/mm^3^	57 (25.4)	121 (58.5)	
≥500 cells/mm^3^	3 (1.3)	69 (33.3)	
